# Optimization of next‐generation sequencing transcriptome annotation for species lacking sequenced genomes

**DOI:** 10.1111/1755-0998.12465

**Published:** 2015-10-14

**Authors:** Nina F. Ockendon, Lauren A. O'Connell, Stephen J. Bush, Jimena Monzón‐Sandoval, Holly Barnes, Tamás Székely, Hans A. Hofmann, Steve Dorus, Araxi O. Urrutia

**Affiliations:** ^1^ Department of Biology and BiochemistryUniversity of BathBathBA2 7AYUK; ^2^ Milner CentreUniversity of BathBathBA2 7AYUK; ^3^ FAS Centre for Systems BiologyHarvard UniversityCambridgeMA02138USA; ^4^ Center for Computational Biology and BioinformaticsDepartment of Integrative BiologyThe University of TexasAustinTX78712USA; ^5^ Department of BiologySyracuse UniversitySyracuseNY13244USA; ^6^ Present address: The Roslin InstituteUniversity of EdinburghEaster BushMidlothianEH25 9RGUK

**Keywords:** *Drosophila*, gene ontology, nonmodel species, primate, RNA‐seq, transcriptome assembly

## Abstract

Next‐generation sequencing methods, such as RNA‐seq, have permitted the exploration of gene expression in a range of organisms which have been studied in ecological contexts but lack a sequenced genome. However, the efficacy and accuracy of RNA‐seq annotation methods using reference genomes from related species have yet to be robustly characterized. Here we conduct a comprehensive power analysis employing RNA‐seq data from *Drosophila melanogaster* in conjunction with 11 additional genomes from related *Drosophila* species to compare annotation methods and quantify the impact of evolutionary divergence between transcriptome and the reference genome. Our analyses demonstrate that, regardless of the level of sequence divergence, direct genome mapping (DGM), where transcript short reads are aligned directly to the reference genome, significantly outperforms the widely used *de novo* and guided assembly‐based methods in both the quantity and accuracy of gene detection. Our analysis also reveals that DGM recovers a more representative profile of Gene Ontology functional categories, which are often used to interpret emergent patterns in genomewide expression analyses. Lastly, analysis of available primate RNA‐seq data demonstrates the applicability of our observations across diverse taxa. Our quantification of annotation accuracy and reduced gene detection associated with sequence divergence thus provides empirically derived guidelines for the design of future gene expression studies in species without sequenced genomes.

## Introduction

Next‐generation transcriptome sequencing (RNA‐seq) has transformed global analyses of gene expression by overcoming the limitations of microarray platforms, including most importantly transcriptional characterization in species yet to have sequenced genomes (Wang *et al*. 2009; Wilhelm & Landry 2009). These species often represent interesting ecological or behavioural model systems, where transcriptome profiling can provide valuable insights into the molecular and physiological underpinnings of complex phenotypic traits. As RNA‐seq data are not dependent on a predefined set of probes corresponding to a particular set of genes, as is the case with microarrays, they have been used in transcriptome profiling of species lacking sequenced genomes where transcriptome annotation is performed using the genome of a related species as a reference (Toth *et al*. 2007; Collins *et al*. 2008; Dassanayake *et al*. 2009; Crawford *et al*. 2010; Künstner *et al*. 2010; Wolf *et al*. 2010; Colgan *et al*. 2011; Esteve‐Codina *et al*. 2011; Garg *et al*. 2011; Kawahara‐Miki *et al*. 2011). However, when annotating transcriptomes using other species' genome as a guide, both RNA‐seq and microarray approaches suffer in terms of accuracy, as sequence divergence between the genome of the species analysed and the one being used as reference impacts on the accuracy of transcript alignment in the case of RNA‐seq data as well as on RNA hybridization with probes when using microarray technology (Renn *et al*. 2004; Machado *et al*. 2009). Indeed, a study using microarray transcriptome profiling measuring mRNA abundance in several *Drosophila* species using *Drosophila melanogaster* as a reference resulted in a diminishing number of orthologous genes detected with increasing sequence divergence and was found to lose its utility at <92% sequence identity, even if correction procedures were applied (Renn *et al*. 2010).

**Figure 1:**
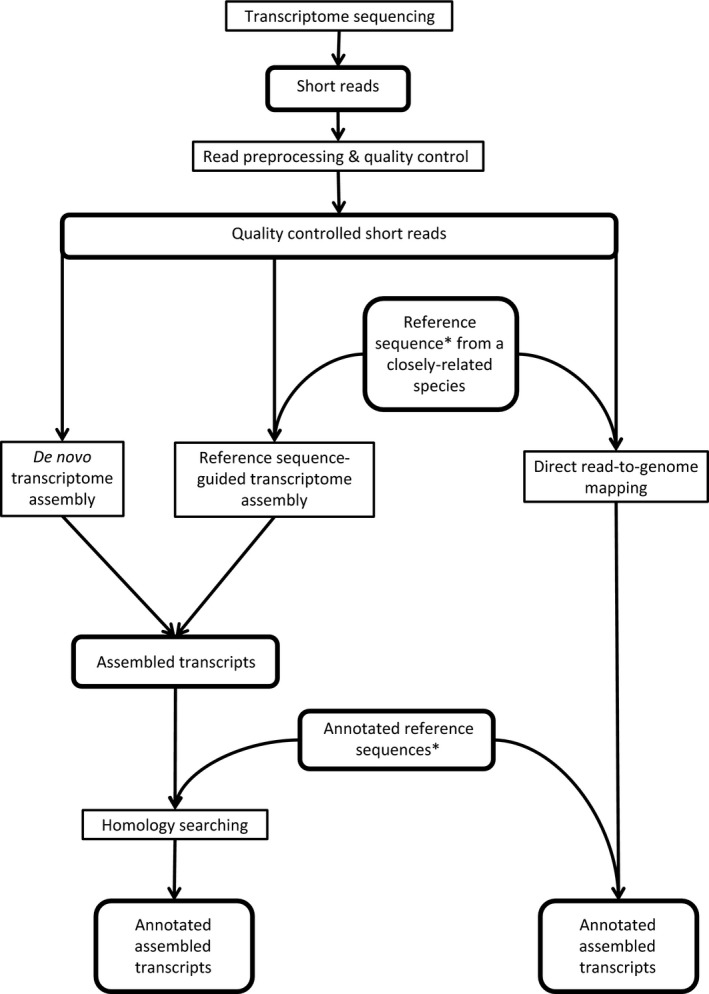
Flow chart outlining pipelines for transcriptome annotation. *De novo* and reference sequence‐guided transcriptome assembly strategies are shown alongside a simpler direct read‐to‐genome mapping approach where quality‐controlled short transcriptome reads are aligned directly against the closest available annotated reference sequence. *Reference sequences used to guide transcriptome assembly or to map reads directly onto may or may not be annotated. If they are not annotated, further information is required providing the coordinates of genomic features of interest. Boxes with squared corners indicate processes; boxes with rounded corners indicate data sets.

In the case of RNA‐seq, annotation of transcriptome data for species that lack sequenced genomes has been carried out using methodologies already employed in the annotation of transcriptomes from species with sequenced genomes. These strategies generate an annotated transcriptome by assembling short transcript reads into larger transcripts or contigs which can then be aligned to known genes in reference genomes. The assembly of short reads into contigs is carried out in two alternative ways: (i) a ‘guided’ assembly that involves aligning short reads to either a reference genome or set of annotated transcripts from a closely related species and then assembling them into contigs by looking for overlaps in the alignment coordinates or (ii) a ‘*de novo*’ assembly that involves aligning short reads to one another, building contigs from the overlaps identified between the sequences; in this case, no additional sequences are used as a guide (Garber *et al*. 2011). Fig. 1 outlines these approaches. Most studies, whether using guided or *de novo* assembly, ultimately aim to annotate assembled transcripts to genes previously annotated in other species to generate hypotheses about the likely function of individual genes and the overall set of functions represented in the genome. This annotation involves the alignment of assembled transcripts to a reference genome, which, when using a reference genome from a different species, is thus affected by increased sequence divergence and leads to a reduction in transcripts that align to known genes (Colgan *et al*. 2011; Kawahara‐Miki *et al*. 2011; Shi *et al*. 2011; Balakrishnan *et al*. 2013; Moghadam *et al*. 2013).

Furthermore, in addition to reductions in the proportion of transcripts which can be annotated to genes, it is likely that as sequence divergence between the species being annotated and the one used as reference increases, the accuracy of transcripts aligned should also decrease. Using *de novo* assembly methods on human transcriptome data, Hornett & Wheat (2012) reported that using increasingly divergent primate and mammalian genomes as references in the annotation of transcripts constructed from either longer 454 transcript sequences or the shorter Illumina reads resulted in an increased rate of error in the annotation of transcripts in addition to shifts in the representation of functional gene annotation terms in the recovered transcriptome. Vijay *et al*. (2013) explored transcriptome annotation in nonmodel species by comparing the performance of *de novo* and guided assemblies constructed from simulated transcript reads aligned to reference transcriptomes with a range of divergence levels (Vijay *et al*. 2013). They found that, when considering the proportion of the reference transcriptome recovered in the simulated assemblies, guided assemblies performed better than *de novo* assemblies with up to 15% sequence divergence, including a minimal reduction in accuracy. Furthermore, when annotating assembled contigs with gene identities, both *de novo* and guided assemblies exhibited increasing error with increasing sequence divergence, and the use of a subset of tissue‐specific genes resulted in misassignment even in the absence of divergence. Lu *et al*. (2013) compared *de novo* and guided assembly methods and demonstrated substantial variability in the performance of different tools. For example, they found that these methods are comparable in terms of the completeness of assembled transcripts, but guided assemblies perform better regarding contiguity (proportion of known transcripts covered by a transcribed sequence fragment), while *de novo* assemblers perform better both in variant resolution and in generating fewer chimeric transcripts.

These previous studies indicate that assembly‐based transcriptome annotation methods are significantly affected by the sequence divergence of the genome used for transcript annotation and also vary in quality depending on the software used. Direct mapping methods, where short reads are not assembled into contigs but instead gene detection is based on short reads aligned directly to the reference genome sequence, as outlined in Fig. 1, have been proposed to allow retention of the fullest possible complement of genomic information for gene identification (Sims *et al*. 2014). However, the potential increase in the number of genes detected could be offset by significantly higher error rates in the assignment of short transcripts to genes as alignment of shorter sequences increases the probability of sequences being erroneously assigned. Also, it is possible that the use of shorter reads for gene identification may result in a substantial proportion of sequences aligning to multiple genomic locations (multimatches) which may again increase ambiguity. Gene detection error rates associated with sequences aligning to single versus multiple locations, and how these rates are impacted by sequence divergence and annotation method, have yet to be determined. Also unexplored are how direct mapping approaches – which are not as widely used – compare to *de novo* and guided assembly methods for annotating transcriptomes using reference genomes at varying levels of divergence. Despite uncertainties about any bias this may introduce, multimatch sequences are often incorporated into transcriptome analyses to increase the quantity of annotated transcripts and genes detected (Mortazavi *et al*. 2008; Brawand *et al*. 2011).

Here we quantitatively assess the impact of sequence divergence between transcriptome and reference species on the performance of a range of next‐generation transcriptome annotation strategies. Using published RNA‐seq data from *D. melanogaster* and genome sequences for 12 *Drosophila* species, the efficacy of two widely used transcript annotation strategies, guided assembly and *de novo* assembly, plus a direct genome mapping (DGM) method which bypasses transcriptome assembly is compared for the first time. The accuracy of gene detection using transcript sequences aligned to single versus multiple locations and biases in gene functional categories associated with each annotation methodology are assessed. Lastly, RNA‐seq data from four primate species are used to confirm the generality of these findings. Our results clearly demonstrate in multiple taxa that the power to accurately recover genes detected as expressed from RNA‐seq data is significantly impacted by the level of divergence between transcriptome and reference species and, more importantly, the annotation method used. We find that, regardless of the level of sequence divergence, DGM significantly outperforms *de novo* and guided assembly‐based strategies in both the quantity and accuracy of gene detection. As such, these results present guidelines for the design of future studies in species without sequenced genomes.

## Materials and methods

### *Drosophila* genome sequences and orthology annotations

Genome releases for *Drosophila melanogaster* (Adams 2000) and 11 additional *Drosophila* species (Richards *et al*. 2005; Clark *et al*. 2007) and orthology relationships were obtained from Flybase (www.flybase.org). Sequence divergence was calculated as the total number of substitutions per site. Pairwise CDS alignments of 12 *Drosophila* species – using the guide tree ((((((dmel,(dsim,dsec)),(dere,dyak)),dana),(dpse,dper)),dwil),((dmoj,dvir),dgri)) – were obtained from ftp://ftp.flybase.net/12_species_analysis/clark_eisen/alignments/ (Clark *et al*. 2007). For each gene, dN/dS was calculated using the Yang and Nielson model, as implemented in the yn00 package of PAML (Yang 2007). Data were filtered to remove sequences of length <150 bp, or with dS < 0.02, dS > 2 or dN > 2 as these can be considered either unreliable for estimates of the dN/dS ratio, unlikely to be bona fide orthologues, or otherwise saturated with substitutions (Löytynoja & Goldman 2008). The divergence of each species from *D. melanogaster* was then considered to be the mean ratio of the number of substitutions to the number of aligned sites per gene. See Table 1 for reference genome species and their respective divergence from *D. melanogaster*, Table S1 (Supporting information) for genome releases and Table S2A (Supporting information ) for gene orthology relationships. Only 1‐to‐1 orthologues were used. Nested and/or overlapping genes were eliminated from all analyses, removing 730 genes from the *D. melanogaster* gene list and 16 from the *D. pseudoobscura* gene list.

**Table 1:** Reference genome species and their respective sequence divergence (total substitutions per site) from the transcriptome species

Reference genome species	Divergence from *Drosophila melanogaster* (total substitutions per site)
A: *Drosophila*	
*Drosophila sechellia*	0.0972
*Drosophila simulans*	0.0952
*Drosophila erecta*	0.2265
*Drosophila yakuba*	0.2149
*Drosophila ananassae*	1.0991
*Drosophila pseudoobscura*	1.1619
*Drosophila persimilis*	1.1705
*Drosophila virilis*	1.1895
*Drosophila grimshawi*	1.2243
*Drosophila mojavensis*	1.2315
*Drosophila willistoni*	1.2268

Reference genome species	Divergence from *H. sapiens*
B: Primates	
*Pan troglodytes*	0.0191
*Gorilla gorilla*	0.0241
*Pongo abelii*	0.0494
*Macaca mulatta*	0.0585

### RNA‐seq data download and preprocessing

Illumina‐derived short reads for the *D. melanogaster* transcriptome were downloaded from the modENCODE database (www.modencode.org, data set 2027: The modENCODE Consortium *et al*. 2011). Short reads (*n *= 9 663 442) were preprocessed in the Penn State Galaxy server (http://galaxyproject.org; Goecks *et al*. 2010; Giardine *et al*. 2005). Reads were groomed into fastqsanger format, sequencing artefacts were removed, and the remaining read set was quality‐filtered using the following criteria: each base was required to satisfy a minimum Phred quality score of 20, equating to a 1% error rate, allowing <10% of the read length (3 bases of 36 base reads) with quality scores below this (Cloonan *et al*. 2008; Crawford *et al*. 2010). This left 6 863 396 reads remaining (71.02% of the original read set).

### Transcriptome annotation through assembly‐based methods

Guided assemblies and a *de novo* assembly were generated using the software packages Velvet Oases and the Columbus extension to velvet (version 1.1 (Zerbino & Birney 2008; Zerbino 2010; Schulz *et al*. 2012). An additional *de novo* assembly was produced using Trinity version r20131110 (Grabherr *et al*. 2011) using the following parameters: –seqType fq –single –min_contig_length 200. For the guided assemblies, alignments between preprocessed *D. melanogaster* reads and the 12 annotated *Drosophila* genomes were performed using the gapped short read alignment program, shrimp version 2.2.0 (Rumble *et al*. 2009; David *et al*. 2011) with default parameters (Table S3, Supporting information), outputting all unaligned reads to the alignment file. For the guided assemblies and the *de novo* assembly constructed with velvet, a multiple k‐mer approach (*k* = 23, 25, 27, 29, 31, 33) was used, generating multiple assemblies based on these k‐mer lengths using the following parameters: ‐reference, ‐short, ‐sam, ‐exp_cov auto, ‐min_contig_lgth 100. Velvet's *mergeAssembly* function was used to merge these multiple k‐mer assemblies. CD‐HIT‐EST (Li & Godzik 2006) was then used to remove contig redundancy that can occur by merging multiple assemblies. Given that redundant contigs can represent alternative splice variants, polymorphisms among the pooled individuals, or sequencing errors, a conservative threshold of 98% sequence similarity was used. All contigs below 100 bp were removed as likely artefacts of the merging and clustering process.

All assemblies were subjected to homology searching using Blast v2.2.26+(Altschul *et al*. 1990), with threshold value E = 1e^−10^, and local alignment against chromosomal databases, as these performed better than coding (CDS) or exon sequences. Significant hits were then verified using fasta36.3.5d (Pearson 2000) with parameters –a (require alignments to use the entire sequence) and ‐A (use Smith–Waterman algorithm).

### Direct genome mapping (DGM) transcript annotatio*n*

Processed *D. melanogaster* RNA‐seq reads were sequentially aligned against each of the 12 *Drosophila* genomes using SHRiMP, as above. Alignments were generated using default parameters (Table S3, Supporting information), and reads were subsequently assigned to genes based on alignment coordinates. Alignments were not filtered by alignment quality.

### Assessment of annotation accurac*y*

Annotation accuracy refers to the correct detection of orthologous annotated reference sequences (genes). Accuracy of the annotation of transcript sequences (contigs or reads) to genes when using a genome sequence from a different species was assessed using the annotation of *D. melanogaster* transcript sequences with the *D. melanogaster* genome as the benchmark. If a gene was detected as expressed in the *D. melanogaster* RNA‐seq data annotated with its own genome and was also detected as expressed when using an alternative genome, then it was considered as correctly detected using that alternative genome.

We compared the accuracy of gene detection for the assembly‐based methods, guided and *de novo*, with DGM. As there is uncertainty over the annotation accuracy of transcripts mapping to multiple genomic locations (Li *et al*. 2010), we explored the accuracy of sequences according to their mapping behaviour. For assembly‐based methods, we segregated contigs that aligned to single genes (single matches) from those that aligned to multiple genes (multimatches). For DGM, we segregated reads that aligned to single genomic locations (single matches) from those that aligned to multiple locations (multimatches). Single matches – often referred to in the literature as uniquely mapped sequences – and multimatches were analysed separately. When a sequence aligns to many locations (multi‐matching), it can have many instances of the same alignment score, introducing ambiguity. To reduce this, we used only those multimatching sequences that had an alignment score higher than all the rest of its other matches: multi‐matching sequences that did not have one alignment scoring higher than the rest were discarded from further analyses.

### Gene functional classification

Genes detected by single‐match reads, using DGM, in *D. melanogaster* were assigned to both the *D. melanogaster* GO slim terms (gene associations [CVS revision 1.220, GOC validation date 24 January 2012]) and the generic GO slim terms [CVS revision 1.864, dated 15 August 2011] obtained from the Gene Ontology Consortium (2000). The proportion of genes associated with each GO slim term and detected with each transcriptome annotation method were calculated. Only those GO slim terms with at least 20 genes annotated in *D. melanogaster* (or *H. sapiens*, see below) were analysed. To assess the loss of annotated genes with the use of increasingly divergent reference genomes, the proportion of genes detected with the use of each reference genome was calculated against the total number of genes identified with each method when using *D. melanogaster* as a reference.

### Primate RNA‐seq

Genome sequences and gene annotations, including GO slim terms, for human and four additional primate species (chimpanzee, gorilla, orangutan and macaque) were downloaded from Ensembl (www.ensembl.org; Flicek *et al*. 2014). Sequence divergence was calculated as the total number of substitutions per site from published data (Chen & Li 2001; Elango *et al*. 2009). See Table 1 for reference genome species and their respective divergence from *H. sapiens*. Orthology annotations were obtained from Brawand *et al*. (2011) – see Table S2B (Supporting information) for orthology relationships. Publicly available single‐end human RNA‐seq data were downloaded from NCBI Sequence Read Archive (www.ncbi.nlm.nih.gov/sra, sample ERS045944). The reads were filtered for sequencing artefacts and subsequently quality‐filtered to the same stringency as the *D. melanogaster* short reads (minimum score of 20 with 10% of the read length [5 bases of 50 base reads] allowed below this), reducing the total number of reads from 29 849 485 to 5 025 987. These were then sequentially directly mapped to each genome. Single‐match mapped reads were extracted and annotated as above. Accuracy was calculated using single‐match human reads assigned to the human genome as benchmark. Gene functional classification according to GO slim terms was performed, as for the *Drosophila* species.

### Statistical analysis

All statistical tests were performed in r, version 3.0.2 (R: A Language and Environment for Statistical Computing 2010). Shapiro–Wilk's tests were used to test for normal data distributions. When all data sets within a given comparison were normally distributed, t‐tests and *F*‐tests were used to test for differences in means and variances, respectively. Where data sets were not consistently normally distributed, comparisons were performed using Mann–Whitney (two‐sample Kruskal–Wallis) tests.

## Results

### Differential impact of sequence divergence on transcript annotation: DGM identifies more genes than alternative strategies

**Figure 2:**
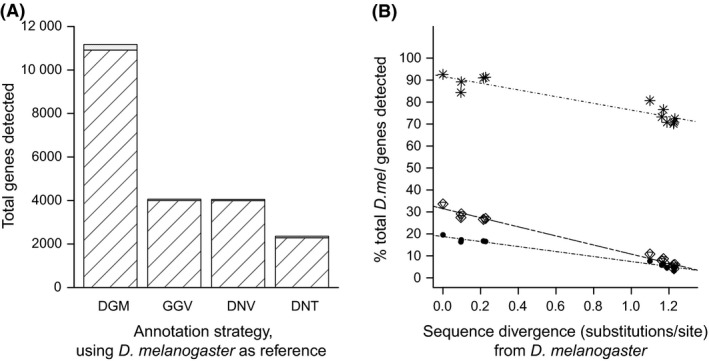
Direct genome mapping (DGM) detects more genes than alternative assembly methods. The efficacy of each transcriptome annotation strategy at recovering genes was assessed using both the same reference species and reference sequences at increasing levels of sequence divergence. (A) Total numbers of genes detected by each strategy (complete stacks) when *Drosophila melanogaster* RNA‐seq sequences are annotated using its own genome. DGM: direct genome mapping; DNT:* de novo* assembly using Trinity; DNV:* de novo* assembly using Velvet Oases; GGV: guided assembly using Velvet Columbus. Genes detected by single‐match sequences are indicated by wide striped sections. (B) The proportion of orthologous genes that are detected (of the total orthologous genes in *Drosophila melanogaster*) at increasing levels of sequence divergence by DGM (stars), guided assemblies (diamonds), and *de novo* assembly using Velvet Oases (inverted triangles) or Trinity (filled circles).

To assess and compare the impact of sequence divergence on the number of genes recovered, that is the sensitivity of each annotation method, by two widely used assembly‐based methods, *de novo* and guided, as well as direct read‐to‐genome mapping (DGM), we annotated *Drosophila melanogaster* next‐generation transcriptome (RNA‐seq) data using its own genome and a further 11 *Drosophila* genomes (Table 1 provides the sequence divergence of these 11 species from *D. melanogaster*). The sensitivity of each annotation method was explored by first establishing a baseline by mapping sequences to the *D. melanogaster* genome. As transcript sequences can map either to a single genomic location or gene (single matches) or to multiple locations or genes (multimatches), we explored gene detection in these different groups. DGM recovered over twice as many genes than any of the assembly‐based methods when annotating the *D. melanogaster* RNA‐seq data with its own genome (Fig. 2A and Table S4, Supporting information): 11 173 genes in total were detected using DGM, whereas 4058 genes were detected in total by the Velvet‐guided assembly and 4051 by the *de novo* assembly. A total of 2361 genes were detected in total by Trinity *de novo* assembly. All methods displayed a reduction in the proportion of genes detected with increasing levels of divergence (Figs 2B and S1, Supporting information). DGM recovered a significantly higher proportion of orthologous genes than any of the assembly methods for each of the 11 genomes analysed (1.308e^−07^ ≤ *P *≤ 3.041e^−06^; Fig. 2B). In contrast, the assembly strategies displayed poorer performance, detecting only 16–34% of orthologous genes in lowly divergent genomes (*D. ananassae* and more closely related species) to below 10% in the more divergent species (Fig. 2B). These results indicate that DGM (i) identifies more genes when using the same genome as reference and (ii) displays superior performance across increasingly divergent genomes, despite the reduction in reads that are mapped in comparison with assembly‐based approaches.

### Increased accuracy of DGM in gene detection

**Figure 3:**
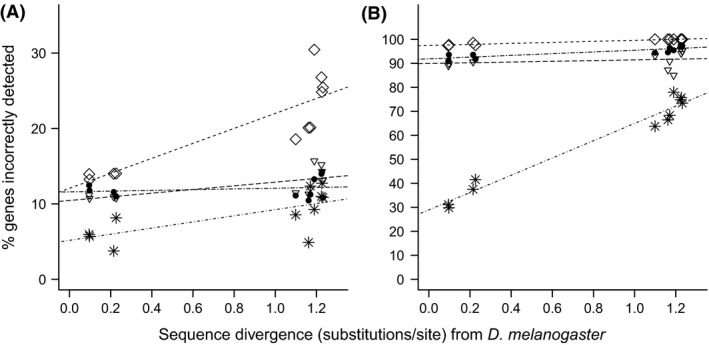
Direct genome mapping (DGM) results in lower gene detection error than alternative assembly methods. Single‐match sequences display significantly lower gene detection error compared to multimatch sequences. The proportion of orthologous genes incorrectly detected by (A) single‐match sequences (unassembled reads or assembled contigs) and (B) multimatch sequences is the lowest for DGM, compared to the assembly methods. Results for DGM (stars), guided assemblies (diamonds), *de novo* assembly using Velvet Oases (inverted triangles) and *de novo* assembly using Trinity (filled circles) are indicated.

To assess annotation error rates as an indication of annotation specificity, gene assignments in the *D. melanogaster* genome were used to quantify the proportion of orthologous genes incorrectly identified using each *Drosophila* genome. Detection error was calculated as the proportion of incorrectly identified genes out of the total number identified. Again, we considered the transcript sequence mapping behaviour, exploring the gene detection accuracy of single matches compared to multimatches. As expected, all annotation strategies were associated with higher error rates with increasing sequence divergence, and multimatch transcripts were associated with markedly higher gene detection error rates compared to single‐match transcripts (Figs 3 and S2, Supporting information). Nonetheless, DGM provided the lowest error rate (6–13%) in gene detection across all species tested, for both single‐match (Fig. 3A; 4.76 × 10^−4^ ≤ *P* ≤ 0.012) and multimatch sequences (Fig. 3B; 4.05 × 10^−4^ ≤ *P* ≤ 4.76 × 10^−4^). Error rates were substantially higher when using both *de novo* assemblies (10–16%) and the guided assemblies (13–30%). It is noteworthy that filtering alignment scores and read counts per gene resulted in marginal improvements in DGM accuracy although this substantially compromised the number of genes detected (Fig. S2, Supporting information), reducing gene detection by approximately 10% for lowly divergent genomes but by more than 30% for the higher divergent genomes (Fig. S2, Supporting information). Hence, using all alignment results is recommended for optimal gene detection. These findings indicate that DGM is significantly more accurate than guided or *de novo* assembly when a corresponding reference genome sequence is unavailable and that this effect is particularly enhanced for multimatch sequences.

### DGM is associated with minimal functional bias in resulting transcriptome annotations

**Figure 4:**
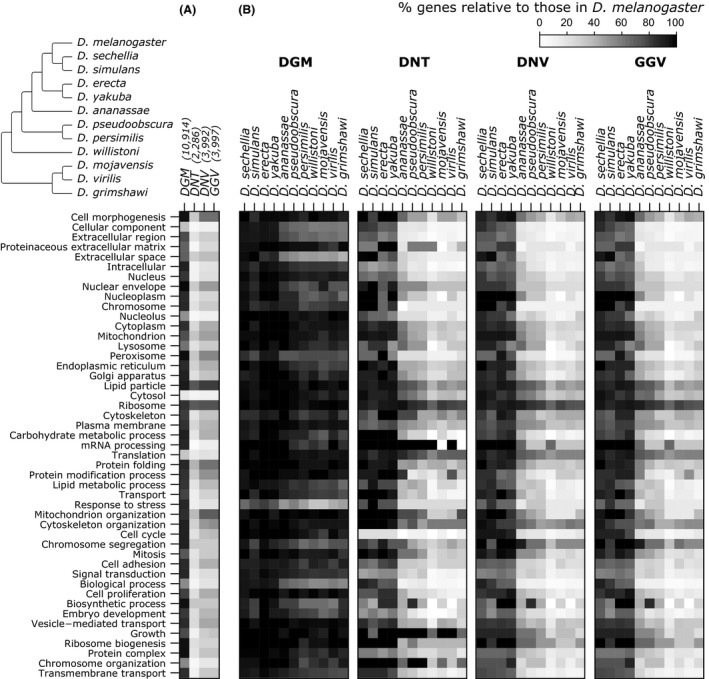
Gene Ontology (GO) annotations for genes detected by different annotation methods. (A) Heatmap of the proportion of genes detected in *Drosophila melanogaster* by different annotation methods (DGM: direct genome mapping, DNT:* de novo* assembly using Trinity, DNV:* de novo* assembly using Velvet Oases, GGV: guided assembly using Velvet Columbus) relative to all protein coding genes in that species. The total number of genes detected with each method in *D. melanogaster* is highlighted between brackets. (B) Heatmap of the proportion of genes detected using each alternative *Drosophila* genome relative to the genes detected in *D. melanogaster* by each annotation method. Colours tending towards black indicate a similar number of genes detected per GO slim term relative to the respective *D. melanogaster* background population, while colours tending towards white indicate a relative decrease in genes assigned per GO slim term (greater depletion). *Drosophila* phylogeny is shown at the top left, as available from Flybase (www.flybase.org).

Due to the nonuniformity of evolutionary rates, transcriptome annotation accuracy using diverged genomes is expected to suffer for rapidly evolving genes (Le Quéré *et al*. 2006) and has a pronounced effect on related Gene Ontology analyses. For example, housekeeping genes tend to evolve more slowly (Duret & Mouchiroud 2000; Lercher *et al*. 2004; Zhang & Li 2004), whereas immune and reproductive genes evolve at a faster rate (Dorus *et al*. 2010). First, we compared the depletion of genes annotated to each GO slim category in the *D. melanogaster* genome when using each transcriptome annotation method with the *D. melanogaster* genome as reference (see Materials and methods). GO slim terms are high‐level Gene Ontology terms which provide a broad overview of the functional content within a set of genes. As expected, given the lower number of genes detected with assembly‐based methods, DGM annotation resulted in a lower depletion of genes annotated to each GO slim term (Fig. 4A). We then compared gene depletion levels for each GO slim term when using increasingly divergent genomes as the reference. The pool of genes detected by each annotation method when using *D. melanogaster* as the reference was taken as the starting point to calculate 100%. We found that DGM was associated with reduced depletion of genes annotated even for the most divergent genomes used (Fig. 4B). Importantly, GO slim terms representing highly conserved functions, such as translation and chromosome organization, display consistently lower levels of depletion (Fig. 4B, and gene detection error, Fig. S3, Supporting information) with increasing divergence of the reference genome (Table S5, Supporting information gives terms with zero gene detection error). Similarly, several GO slim terms associated with rapidly evolving processes, such as reproduction (Dorus *et al*. 2010) and mRNA processing (Marz *et al*. 2008), exhibit consistently high depletion (Fig. 4B) and gene detection error rates (Table S6, Supporting information).

### Corroborating DGM performance in alternative taxa

**Figure 5:**
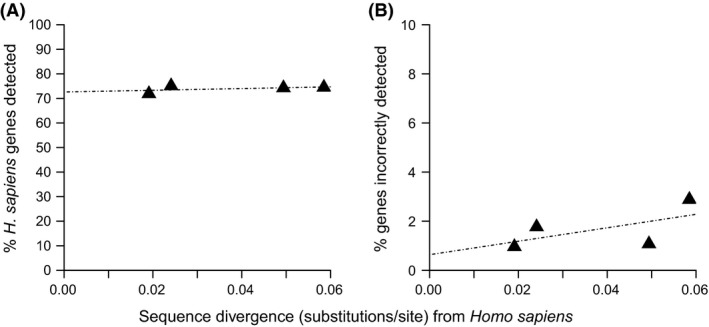
Direct genome mapping (DGM) characteristics in primates. (A) Proportion of human orthologues detected (of the total orthologous genes in human) by single‐match reads when human RNA‐seq reads were mapped to the alternative nonhuman primate genomes. (B) Proportion of orthologous genes incorrectly annotated using the human genome annotation of reads as the benchmark.

To establish whether DGM single‐match read performance is consistent across taxa, our analysis was repeated using human RNA‐seq data sequentially aligned against other primate genomes (see Table 1 for reference genome species and their respective divergence from humans). Single‐match reads were extracted and assigned to orthologous genes, with the numbers of genes detected and respective error rates obtained (Fig. 5). Compared to *Drosophila*, the proportion of orthologous genes detected is slightly lower in primates (approximately 73% at up to 0.06 total substitutions per site divergence from humans for primate species, versus 84–89% at approximately 0.1 total substitutions per site divergence from *D. melanogaster* for the *Drosophila* species). This is likely to be accounted for in part by the greater amounts of repetitive sequence in the primate genomes (Liu *et al*., 2003) and the higher proportion of genes within large, highly homologous gene families. However, the proportion of genes detected across increasingly divergent genomes are well maintained. This is perhaps expected given the low range of divergence among the primate genomes analysed but is nonetheless informative in choosing an alternative reference genome for transcriptome analysis by DGM. Error rates for gene detection are also lower for primates compared to *Drosophila*, a finding that we attribute to the longer read length in primates (primate = 50nt; *Drosophila* = 36nt). Lastly, functional bias in gene detection, as was the case with *Drosophila*, was variable across functional terms (Fig. S4, Supporting information). Consistent with the low divergence of these primate genomes, GO slim terms detected in primates exhibited very low error, especially for those categories with highly conserved functions (Fig. S5, Supporting information).

## Discussion

We have conducted a systematic performance comparison of two assembly‐based methods and direct read‐to‐genome mapping (DGM) when applied to the annotation of transcriptome data for species without sequenced genomes. Short reads for *Drosophila melanogaster* were annotated using 11 other *Drosophila* genomes as the reference to measure the efficiency and accuracy of annotation as a function of nucleotide divergence. Key findings were then validated using primate species. We found that DGM is substantially more effective in gene recovery both when the transcriptome and reference sequences are the same or divergent. Specifically, DGM detects over twice the number of genes relative to the best assembly‐based methods in the absence of divergence; its superior performance increases with divergence. Importantly, we were able to benchmark gene annotation accuracy and assess bias in the detection of gene functional categories: DGM displayed the highest accuracy in gene detection and the lowest depletion of functional categories across wide ranges of divergence. This indicates that DGM is more robust at detecting the functional complexity of transcriptome profiles when there is divergence between transcriptome and reference species, and demonstrates that studies aiming to characterize novel transcriptomes should benefit from this powerful and comparatively low error technique compared to assembly‐based methods. To help inform the design of future comparative functional genomics studies aiming to use multiple transcriptome/reference species with differing divergence levels between them, we assessed the impact of divergence on gene detection error in GO terms. Error differs according to the term with many showing stable error across the divergence levels tested. Similar trends are observed with primate data, and when comparing the two lineages, categories with consistently high (reproduction, and mRNA processing) or low (translation and chromosome organization) error can be observed.

Our observations of decreased gene detection, increased gene detection error and functional bias with increasing divergence of the reference genome with all annotation methods tested are consistent with similar studies using assembly‐based transcriptome annotation methods (Hornett & Wheat 2012; Lu *et al*. 2013; Vijay *et al*. 2013). Indeed, the trends of gene detection and transcript assignment error with increasing divergence of our primate results recapitulate Hornett & Wheat's (2012) findings using assembled primate sequences. It is worth noting, however, that these studies did not assess the performance of transcriptome annotation using direct read‐to‐genome mapping which bypasses the assembly of reads into contigs. Lu *et al*. (2013) advocated an approach integrating aspects of guided and *de novo* assembly methods when there is no sequence divergence between transcriptome and reference species. Our findings do not support this: when comparing *de novo* with guided assembly methods, although these approaches performed comparably for the quantity of genes detected, both of our *de novo* assemblies performed significantly better than the guided assemblies regarding accuracy of gene detection and transcript assignment across large evolutionary distances. Interestingly, despite detecting different numbers of genes when transcriptome and reference species are the same (Velvet/Oases: 4051; Trinity: 2361), the two *de novo* assemblies showed similar levels of accuracy with increasing divergence. The relative number of transcripts produced by each assembly tool (Velvet/Oases: 14 555; Trinity: 5026) suggests that Trinity may be more efficient at constructing valid, longer‐length transcripts. Our findings highlight the importance of assessing the gene detection capabilities as well as the accuracy of the annotations when judging transcriptome annotation techniques as the numbers of transcripts produced and the numbers of genes to which these have homology matches are not necessarily good metrics of assembly quality.

We further show that transcript sequences mapping to a single location or gene are far more accurate than the top‐scoring hits of multimatch sequences. For gene expression studies, it has been noted that multimatch reads should be included to provide more representative expression profiles (Mortazavi *et al*. 2008). Some annotation tools have been developed to help deal with these problematic sequences, such as ERANGE (Mortazavi *et al*. 2008), BM‐MAP (Ji *et al*. 2011), RSEM (Li *et al*. 2010) and SeqEm (Paşaniuc *et al*. 2011). However, our results demonstrate that the inclusion of multimatch reads, or indeed contigs that map to multiple genes, in any transcriptome study of a species lacking a sequenced genome would introduce high levels of error in both transcript sequence assignment and gene detection and hence should be avoided, especially if the divergence between transcriptome and reference species is high. One way to incorporate these approaches, potentially bolstering the gene expression profile to a more representative degree without compromising excessively on accuracy, may be to obtain the list of genes identified by single‐match reads and subsequently incorporate only those multimatch reads that aligned to genes in that list. As sequencing technologies improve, increasing read length will reduce the number of multimatch reads, thereby reducing ambiguity and enabling transcriptomic analysis of a wider repertoire of organisms, with potentially greater evolutionary divergence between themselves and the closest available annotated reference species.

Previous microarray studies using multiple transcriptome/reference species pairs from various taxa have highlighted a key issue: when comparing the transcriptomes of species lacking sequenced genomes that have been annotated using a related genome sequence, the gene lists identified and subsequently compared the need to be standardized (Machado *et al*. 2009; Renn *et al*. 2010). Our results in both *Drosophila* and primate species not only reiterate this issue, highlighting how the choice of annotation strategy influences the degree of function bias, but also demonstrate that common functional categories suffer similarly from gene detection error induced by divergence. This may reflect certain gene categories being associated with similar rates of sequence divergence across metazoan lineages. Particular terms are observed to have consistently higher error rates in both *Drosophila* and primate species, such as reproduction and mRNA processing. This may be explained by a number of factors, including comparatively high rates of evolution (Dorus *et al*. 2010), gene duplication and rapid synteny changes (Marz *et al*. 2008) operating on such types of genes, but also lineage‐specific changes in exon usage via differentially regulated alternative splicing (Blekhman *et al*. 2010). However, this may also be contributed to by inconsistent Gene Ontology annotations across the range of species used (Khatri & Drăghici 2005), particularly where the Gene Ontology annotations include multiple terms.

**Figure 6:**
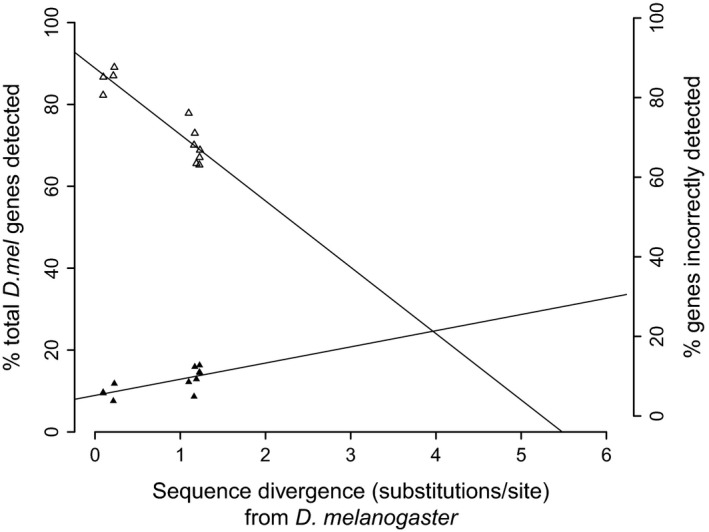
Impact of sequence divergence on gene detection and misidentification rates using direct genome mapping (DGM): a reference guide for future studies. Trend lines display the proportion of *Drosophila* reference species genes detected by single‐match reads (open triangles) and associated errors rates (filled triangles) as a function of sequence divergence. Detection and error rates are estimated to be comparable at approximately 4.0 substitutions per site. The relationship between the efficacy of detection and inherent misidentification of genes can be of use to investigators in future experimental design and data analysis if they have an accurate estimation of nucleotide divergence between their study species and the reference genome they are utilizing.

We expect that our findings regarding gene detection capabilities and error, functional bias, and the manner with which these are exacerbated with increasing nucleotide divergence will aid the interpretation of transcriptome annotation using species lacking sequenced genomes in comparative analyses, particularly where multiple species pairs are to be compared. Using a reference species with the lowest possible nucleotide divergence from the transcriptome species and only utilizing single‐match reads from DGM is recommended for gene detection studies. If the relationships of both gene detection and gene detection error with sequence divergence are linear, as we have assumed them to be, this enables us to illustrate thresholds of divergence between transcriptome and reference species below which gene detection is maximized while gene detection error remains low (Fig. 6). We observe a similar pattern with the primate data where the crossover between detection and error is shifted to a lower region of sequence divergence. This is most likely due to the low sequence divergence between the species used and relatively few data points (data not shown). Together, this illustrates how factors, such as divergence between transcriptome and reference species, and genomic features, such as complexity, repetitive sequence and gene length, can impact on the power to accurately recover genes. Additionally, as high levels of divergence can lead to the enrichment of slow evolving, highly conserved genes overpowering depleted fast‐evolving genes, using multiple transcriptome/reference species pairs with varying degrees of divergence between them may lead to noncomparable results. As such, the selection of species with no available genome sequence should be based on the availability of the closest possible reference sequence and consider the knowledge base surrounding that reference sequence. Although testing the limits of the quality of reference genome needed for transcriptome annotation is beyond the scope of this study, given that the coverage, assembly and annotation of genomes of the species covered in this study are of variable quality, our results suggest that the use of the closest available sequenced relative might be preferable to using a more distant species with a higher‐quality genome at least based on quality variability covered in this study.

## Conclusions

Our results demonstrate that, compared to the conventionally used assembly‐based methods of *de novo* and guided assembly, DGM has superior performance when annotating the transcriptome of a species without a sequenced genome, if using the annotated reference sequence from a closely related species. Importantly, DGM is also associated with the greatest accuracy and a minimal loss of gene detection over large evolutionary distances and recovers a more representative functional profile (as assessed by GO slim categories) of genes than the other strategies.

Compared to the assembly‐based methods, DGM is a very simple process to employ: it requires few steps and a small amount of ‘hands‐on’ time to implement – it requires no optimization, except for establishing the user's preferred levels of short read preprocessing and alignment parameters, and no subsequent homology searching. Together, our findings pave the way for the utilization of a wide variety of nonmodel species in transcriptome studies where the closest available reference species is not necessarily a close relative.

N.F.O., H.A.H., T.S. and A.O.U. conceived the study. NFO, LAO, SD and AOU designed analyses with additional input from all authors. N.F.O., L.A.O., S.J.B., J.M.S. and H.B. performed all analyses presented. N.F.O. wrote all Python scripts for D.G.M. and accuracy assessments of all strategies tested. N.F.O. and J.M.S. performed functional analyses on all *Drosophila* and primate data. L.A.O. generated all assemblies used. S.J.B. performed all homology searching. H.B. performed all primate D.G.M. N.F.O., S.D. and A.O.U. wrote manuscript with contributions from all authors.

## Data Accessibility

*Drosophila melanogaster* RNA‐seq sequences: http://data.modencode.org/; data set 2027.*Drosophila* reference genome sequences:

*D. melanogaster*: ftp://ftp.flybase.net/genomes/Drosophila_melanogaster/dmel_r5.41_FB2011_09/fasta/dmel-all-chromosome-r5.41.fasta.gz

*Drosophila sechellia*: ftp://ftp.flybase.net/genomes/Drosophila_sechellia/dsec_r1.3_FB2011_08/fasta/dsec-all-chromosome-r1.3.fasta.gz

*Drosophila simulans*: ftp://ftp.flybase.net/genomes/Drosophila_simulans/dsim_r1.3_FB2011_08/fasta/dsim-all-chromosome-r1.3.fasta.gz

*Drosophila yakuba*: ftp://ftp.flybase.net/genomes/Drosophila_yakuba/dyak_r1.3_FB2011_02/fasta/dyak-all-chromosome-r1.3.fasta.gz

*Drosophila erecta*: ftp://ftp.flybase.net/genomes/Drosophila_erecta/dere_r1.3_FB2011_07/fasta/dere-all-chromosome-r1.3.fasta.gz

*Drosophila ananassae*: ftp://ftp.flybase.net/genomes/Drosophila_ananassae/dana_r1.3_FB2011_02/fasta/dana-all-chromosome-r1.3.fasta.gz

*Drosophila pseudoobscura*: ftp://ftp.flybase.net/genomes/Drosophila_pseudoobscura/dpse_r2.24_FB2011_09/fasta/dpse-all-chromosome-r2.24.fasta.gz

*Drosophila persimilis*: ftp://ftp.flybase.net/genomes/Drosophila_persimilis/dper_r1.3_FB2010_02/fasta/dper-all-chromosome-r1.3.fasta.gz

*Drosophila willistoni*: ftp://ftp.flybase.net/genomes/Drosophila_willistoni/dwil_r1.3_FB2010_02/fasta/dwil-all-chromosome-r1.3.fasta.gz

*Drosophila mojavensis*: ftp://ftp.flybase.net/genomes/Drosophila_mojavensis/dmoj_r1.3_FB2011_05/fasta/dmoj-all-chromosome-r1.3.fasta.gz

*Drosophila virilis*: ftp://ftp.flybase.net/genomes/Drosophila_virilis/dvir_r1.2_FB2011_07/fasta/dvir-all-chromosome-r1.2.fasta.gz

*Drosophila grimshawi*: ftp://ftp.flybase.net/genomes/Drosophila_grimshawi/dgri_r1.3_FB2010_02/fasta/dgri-all-chromosome-r1.3.fasta.gz

Human RNA‐seq sequences: http://www.ncbi.nlm.nih.gov/sra/?term=ERS045944

Primate reference genome sequences: *Pan troglodytes*: ftp://ftp.ensembl.org/pub/release-68/fasta/pan_troglodytes/dna/Pan_troglodytes.CHIMP2.1.4.68.dna.toplevel.fa.gz

*Gorilla gorilla*: ftp://ftp.ensembl.org/pub/release-68/fasta/gorilla_gorilla/dna/Gorilla_gorilla.gorGor3.1.68.dna.toplevel.fa.gz

*Pongo abelii*: ftp://ftp.ensembl.org/pub/release-68/fasta/pongo_abelii/dna/Pongo_abelii.PPYG2.68.dna.toplevel.fa.gz

*Macaca mulatta*: ftp://ftp.ensembl.org/pub/release-68/fasta/macaca_mulatta/dna/Macaca_mulatta.MMUL_1.68.dna.toplevel.fa.gz

Primate gene annotations: http://www.ensembl.org/biomart/martview/

Genome assemblies and alignments, and raw data underlying figures: Dryad doi:10.5061/dryad.2v3 h4

## Supporting information


**Table S1** Genome sequence versions.
**Table S2** Drosophila and primate genes orthology relationships.
**Table S3** SHRiMP default parameters used for short read alignment.
**Table S4** Orthologous gene detection using alternative annotation strategies for total sequences (total) and single‐match sequences (SM).
**Table S5** GO slim terms with zero gene detection error for *Drosophila* and primate species.
**Table S6** Top 20% of GO slim terms ranked by gene detection error for *Drosophila* and primate species.
**Fig. S1** Direct genome mapping displays lower gene detection error than alternative assembly methods ‐ trend recapitulated when results are plotted against divergence in MYA.
**Fig. S2** Increased DGM annotation accuracy using reads filtered for low alignment scores and higher read counts per gene.
**Fig. S3** Gene detection error varies with functional gene category.
**Fig. S4** Gene Ontology (GO) annotations for genes detected by using primate data.
**Fig. S5** Gene detection error varies with functional gene category in primate species.
