# Supplementary material for: Optimization of next‐generation sequencing transcriptome annotation for species lacking sequenced genomes
Source: Mol Ecol Resour. 2015 Oct 14;16(2):446–58. doi: 10.1111/1755-0998.12465 (PMC4982090; doi:10.1111/1755-0998.12465)
Supplement: Supplementary file 1 — Table S1 Genome sequence versions. Table S2 Drosophila and primate genes orthology relationships. Table S3 SHRiMP default parameters used for short read alignment. Table S4 Orthologous gene detection using alternative annotation strategies for total sequences (total) and single‐match sequences (SM). Table S5 GO slim terms with zero gene detection error for Drosophila and primate species. Table S6 Top 20% of GO slim terms ranked by gene detection error for Drosophila and primate species. Fig. S1 Direct genome mapping displays lower gene detection error than alternative assembly methods ‐ trend recapitulated when results are plotted against divergence in MYA. Fig. S2 Increased DGM annotation accuracy using reads filtered for low alignment scores and higher read counts per gene. Fig. S3 Gene detection error varies with functional gene category. Fig. S4 Gene Ontology (GO) annotations for genes detected by using primate data. Fig. S5 Gene detection error varies with functional gene category in primate species. [file MEN-16-446-s001.docx]

## Supplementary Tables

Table S1: Genome sequence versions.

| **Genome sequences** | **Release details*** |
| --- | --- |
| *Drosophila melanogaster* | 5.41 |
| *Drosophila ananassae* | 1.3 |
| *Drosophila erecta* | 1.3 |
| *Drosophila grimshawi* | 1.3 |
| *Drosophila mojavensis* | 1.3 |
| *Drosophila persimilis* | 1.3 |
| *Drosophila pseudoobscura* | 2.24 |
| *Drosophila sechellia* | 1.3 |
| *Drosophila simulans* | 1.3 |
| *Drosophila virilis* | 1.2 |
| *Drosophila willistoni* | 1.3 |
| *Drosophila yakuba* | 1.3 |
| *Homo sapiens* | 68 |
| *Pan troglodytes* | 68 |
| *Gorilla gorilla* | 68 |
| *Pongo abelii* | 68 |
| *Macaca mulatta* | 68 |

* Fly genome sequences downloaded from Flybase (www.flybase.org) and primate genomes downloaded from Ensembl ([www.ensembl.org](http://www.ensembl.org); Flicek et al., 2014).

Table S2: Drosophila and primate genes orthology relationships (see separate spreadsheet: Tables S2 and S5.xlsx).

Table S3: SHRiMP default parameters used for short read alignment. Please refer to the SHRiMP documentation for further details (http://compbio.cs.toronto.edu/shrimp/).

| **Parameter** | **Default value** |
| --- | --- |
| Spaced Seeds | 11110111101111, 1111011100100001111, 1111000011001101111 |
| Maximum Hits per Read | 10 |
| Maximum alignments per read | All |
| Match Window Length | 140.00% |
| Match Mode | Unpaired |
| Smith-Waterman Match Score | 10 |
| SW Mismatch Score | -15 |
| SW Gap Open Score (Reference) | -33 |
| SW Gap Open Score (Query) | -33 |
| SW Gap Extend Score(Reference) | -7 |
| SW Gap Extend Score (Query) | -3 |
| Window Generation Threshold | 55.00% |
| SW Full Hit Threshold | 50.00% |
| Number of Threads | 1 |
| Paired Mode | None |
| Minimum and Maximum Insert Size | 0,1000 |
| Maximum read length | 1000 |
| Perform Ungapped Alignment | disabled |
| Perform full global alignment | enabled |
| Perform local alignment | disabled |
| Trim trailing B qual values | disabled |
| Negative Strand Alignment Only | disabled |
| Positive Strand Alignment Only | disabled |

Table S4. Orthologous gene detection using alternative annotation strategies for total sequences (total) and single-match sequences (SM). Divergence given as both sequence divergence (total substitutions, ‘subst’) and million years ago (‘MYA’).

| **Species** | **DGM** | **Guided assembly**  **(Velvet Columbus)** | ***de novo* assembly (Velvet Oases)** | ***de novo* assembly (Trinity)** |
| --- | --- | --- | --- | --- |
| *D. melanogaster* (0subst, 0MYA) | 11173 (total), 10914 (SM) | 4058 (total), 3997 (SM) | 4051 (total), 3992 (SM) | 2361 (total), 2286 (SM) |
| *D. sechellia* (0.097subst, 5.4MYA) | 10768 (total), 10460 (SM) | 3515 (total), 3428 (SM) | 3523 (total), 3438 (SM) | 2086 (total), 1981 (SM) |
| *D. simulans* (0.095subst, 5.4MYA) | 10181 (total), 9926 (SM) | 3313 (total), 3233 (SM) | 3319 (total), 3233 (SM) | 1978 (total), 1884 (SM) |
| *D. yakuba* (0.227subst, 12.8MYA) | 11015 (total), 10749 (SM) | 3263 (total), 3142 (SM) | 3275 (total), 3153 (SM) | 2000 (total), 1901 (SM) |
| *D. erecta* (0.215subst, 12.6MYA) | 10981 (total), 10500 (SM) | 3211 (total), 3109 (SM) | 3214 (total), 3116 (SM) | 2023 (total), 1902 (SM) |
| *D. ananassae* (1.613subst, 44.2MYA) | 9738 (total), 9396 (SM) | 1308 (total), 1265 (SM) | 1307 (total), 1268 (SM) | 918 (total), 846 (SM) |
| *D. pseudoobscura* (1.861subst, 54.9MYA) | 8835 (total), 8456 (SM) | 961 (total), 925 (SM) | 965 (total), 931 (SM) | 701 (total), 646 (SM) |
| *D. persimilis* (1.899subst, 54.9MYA) | 9242 (total), 8806 (SM) | 1051 (total), 1010 (SM) | 1060 (total), 1012 (SM) | 772 (total), 701 (SM) |
| *D. willistoni* (2.744subst, 62.2MYA) | 8542 (total), 7913 (SM) | 678 (total), 608 (SM) | 678 (total), 604 (SM) | 558 (total), 418 (SM) |
| *D. mojavensis* (2.528subst, 62.9MYA) | 8459 (total), 7870 (SM) | 705 (total), 618 (SM) | 715 (total), 619 (SM) | 602 (total), 459 (SM) |
| *D. virilis* (2.263subst, 62.9MYA) | 8741 (total), 8309 (SM) | 735 (total), 620 (SM) | 718 (total), 623 (SM) | 567 (total), 436 (SM) |
| *D. grimshawi* (2.297subst, 62.9MYA) | 8550 (total), 8092 (SM) | 500 (total), 439 (SM) | 484 (total), 435 (SM) | 388 (total), 319 (SM) |

Table S5. GO slim terms with zero gene detection error for *Drosophila* and primate species (see separate spreadsheet: ‘Tables S2 and S5.xlsx’).

Table S6. Top 20% of GO slim terms ranked by gene detection error for *Drosophila* and primate species. The terms reproduction, biosynthetic process, and mRNA processing are highlighted (bold italic) as they exhibit consistently high error in all species tested, both *Drosophila* and primate.

(A)

| ***Drosophila*** | **GO slim term** | **Mean error** |
| --- | --- | --- |
|  | ***reproduction*** | 0.666666667 |
|  | sulfur compound metabolic process | 0.583333333 |
|  | cytoplasmic membrane-bounded vesicle | 0.2757935 |
|  | microtubule organizing center | 0.273015667 |
|  | lysosome | 0.175490333 |
|  | cell differentiation | 0.1722 |
|  | cell-cell signaling | 0.1524 |
|  | proteinaceous extracellular matrix | 0.1298191 |
|  | plasma membrane organization | 0.1170635 |
|  | DNA metabolic process | 0.116666667 |
|  | ***mRNA processing*** | 0.1142055 |
|  | locomotion | 0.108670067 |
|  | nucleolus | 0.09453935 |
|  | biological process | 0.0918 |
|  | cell proliferation | 0.0916 |
| **Primates** | external encapsulating structure | 0.25 |
|  | hydrolase activity, acting on carbon-nitrogen (but not peptide) bonds | 0.062665375 |
|  | structural constituent of ribosome | 0.05800995 |
|  | ribosome | 0.041368375 |
|  | protein folding | 0.038986275 |
|  | translation factor activity, nucleic acid binding | 0.0388671 |
|  | unfolded protein binding | 0.0381215 |
|  | generation of precursor metabolites and energy | 0.031932935 |
|  | translation | 0.03125575 |
|  | nuclear chromosome | 0.02761715 |
|  | mitochondrion | 0.02669375 |
|  | growth | 0.0266444 |
|  | ATPase activity | 0.02652197 |
|  | nucleobase-containing compound catabolic process | 0.02559745 |
|  | cellular amino acid metabolic process | 0.025241675 |
|  | oxidoreductase activity | 0.02487455 |
|  | cilium | 0.02313055 |
|  | chromosome | 0.02187006 |
|  | nucleic acid binding transcription factor activity | 0.02138506 |
|  | nucleocytoplasmic transport | 0.021334923 |
|  | symbiosis, encompassing mutualism through parasitism | 0.020329433 |
|  | transmembrane transporter activity | 0.020212498 |
|  | biosynthetic process | 0.019870675 |
|  | ***reproduction*** | 0.01971535 |
|  | ***mRNA processing*** | 0.01964495 |
|  | cellular nitrogen compound metabolic process | 0.018890025 |
|  | nucleoplasm | 0.0186067 |

## Supplementary Figures

**Fig. S1.** **Direct genome mapping displays lower gene detection error than alternative assembly methods - trend recapitulated when results are plotted against divergence in MYA.** (A) The proportion of orthologous genes detected incorrectly by single-match sequences (unassembled reads or assembled contigs) was the lowest for direct genome mapping, compared to the assembly methods. (B) The proportion of orthologous genes detected incorrectly by multi-match sequences was the lowest for direct genome mapping, compared to the assembly methods. Single-match sequences displayed significantly lower gene detection error compared to multi-match sequences. Results for direct genome mapping (stars), genome-guided assemblies (diamonds), *de novo* assembly using Velvet/Oases (inverted triangles), and *de novo* assembly using Trinity (filled circles) are indicated.

**Fig. S2. Increased DGM annotation accuracy using reads filtered for low alignment scores and higher read counts per gene.** Given that DGM performed the best for gene detection, gene detection accuracy was explored in greater depth. Allocating reads to bins according to score, reads with the lowest score range (< 199) were significantly different from the others in terms of the proportion of reads in that bin that were correctly assigned (ANOVA: p < 2.2e^-16^, Tukey HSD test: p < 2.2e^-16^ for ‘<199 score’ bin compared to all others). Similarly, when allocating genes to bins according to read count, genes with less than 5 reads assigned were significantly different from the others in terms of the proportion of genes in that bin that were correctly detected (ANOVA: p < 2.2e^-16^, Tukey HSD test: p < 2.2e^-16^ for ‘<5 reads per gene’ bin compared to all others). Hence, data for genes detected by single-match reads were filtered to, firstly, remove reads with an alignment score of less than 199, and, secondly, remove genes with fewer than 5 reads assigned. This caused a moderate drop in the proportion of orthologous *D. melanogaster* genes that can be detected (A) and improves DGM accuracy by a small amount (B), particularly at high levels of divergence. (A) Open triangles represent the proportions of *D. melanogaster* genes detected by all reads using each alternative genome; filled triangles represent the proportions of *D. melanogaster* genes detected by single-match reads using each alternative genome; stars represent the proportions of *D. melanogaster* genes detected by single-match reads using each alternative genome after filtering reads for low alignment score and genes for low read counts per gene. (B) Filled triangles represent the proportions of *D. melanogaster* genes detected incorrectly by single-match reads using each alternative genome; stars represent the proportions of *D. melanogaster* genes detected incorrectly with single-match reads using each alternative genome after filtering reads for low alignment score and genes for low read counts per gene.

**Fig. S3. Gene detection error varies with functional gene category.** Mean error scores of gene detection using DGM per GO slim term for (A) *D. sechellia* and *D. simulans*, (B) *D. erecta* and *D. yakuba*, and (C) *D. pseudoobscura* and *D. persimilis* were plotted (employing a minimum threshold of 20 *D. melanogaster* genes per GO slim term). Particular GO slim terms show heightened mean error scores across all levels of divergence, such as lysosome, whereas other terms maintain low levels of error, such as translation.

**Fig. S4. Gene Ontology (GO) annotations for genes detected by using primate data.** For ease of viewing the results have been split into panels 1 and 2: panel 2 is simply a continuation of the data set from panel 1. A) Heatmap of the proportion of genes detected in *H. sapiens* by direct genome mapping (DGM) relative to all protein coding genes in that species. The total number of genes detected by DGM in *H. sapiens* is highlighted between brackets. B) Heatmap of the proportion of genes detected using each alternative primate genome relative to the genes in *H. sapiens* detected by DGM. Colours tending towards black indicate similar number of genes detected per GO slim term relative to the respective *H. sapiens* background population, while colours tending towards white indicate a relative decrease of genes assigned to a particular GO slim term when using each of the alternative primate genomes.

orangutan

**Fig. S5. Gene detection error varies with functional gene category in primate species.** Mean error scores of gene detection using DGM per GO slim term for (A) chimpanzee, (B) gorilla, (C) orangutan, and (D) macaque were plotted employing a minimum threshold of 20 human genes per GO slim term and selecting the 50 terms with highest error per species. Particular GO slim terms show heightened mean error scores across all levels of divergence, such as external encapsulating structure.
